# Neurological Complications in a Patient With Thalassemia Intermedia and Chronic Myeloid Leukemia: A Case of Extramedullary Hematopoiesis-Induced Spinal Cord Compression

**DOI:** 10.7759/cureus.84844

**Published:** 2025-05-26

**Authors:** Ozlem Onder, Sahin Isik, Ersen Ceylan

**Affiliations:** 1 Department of Neurology, Near East University, Nicosia, CYP; 2 Department of Radiation Oncology, Near East University, Nicosia, CYP

**Keywords:** chronic myeloid leukemia, extramedullary hematopoiesis, myelopathy, spinal cord compression, thalassemia intermedia

## Abstract

Extramedullary hematopoiesis (EMH) is a rare but significant compensatory response to chronic ineffective erythropoiesis, frequently seen in hematologic disorders such as thalassemia intermedia (TI) and chronic myeloid leukemia (CML). While EMH is typically asymptomatic, it can manifest as paraspinal masses that cause spinal cord compression, resulting in severe neurological deficits. This case highlights the complexities in managing overlapping hematologic conditions and emphasizes the importance of timely diagnosis and intervention. We present a 50-year-old male with a longstanding history of TI and CML, who developed progressive lower extremity weakness, gait instability, and sensory deficits due to spinal cord compression from EMH. This case underscores the critical need for clinical awareness and early intervention in patients with hematologic disorders who present with neurological symptoms. EMH-induced spinal cord compression, though rare, can lead to significant morbidity if left untreated. Multidisciplinary management involving neurology, hematology, radiology, and rehabilitation is essential to optimize outcomes and prevent permanent neurological deficits in affected patients.

## Introduction

Extramedullary hematopoiesis (EMH) is a compensatory response to chronic ineffective erythropoiesis and is commonly observed in conditions such as thalassemia intermedia (TI) and chronic myeloid leukemia (CML) [1]. While the bone marrow typically serves as the primary site for hematopoiesis, when its capacity is exceeded or ineffective, hematopoietic activity can extend to extramedullary sites, including the spleen, liver, and paravertebral regions [2].

In rare cases, EMH can manifest as paraspinal masses, leading to spinal cord compression and subsequent neurological deficits [3]. Spinal EMH is often asymptomatic but may present with severe neurological symptoms if the spinal canal is significantly compromised [4]. This is especially concerning in patients with coexisting conditions that exacerbate the hematopoietic burden, such as CML, where the increased demand for blood cell production further stresses the hematopoietic system [5].

We present a case of a 50-year-old male with TI and CML who developed progressive neurological symptoms due to spinal cord compression from extensive EMH. This case illustrates the complexity of managing overlapping hematological disorders and emphasizes the critical need for prompt diagnosis and intervention to address potentially reversible neurological deficits.

## Case presentation

**Figure 1:**
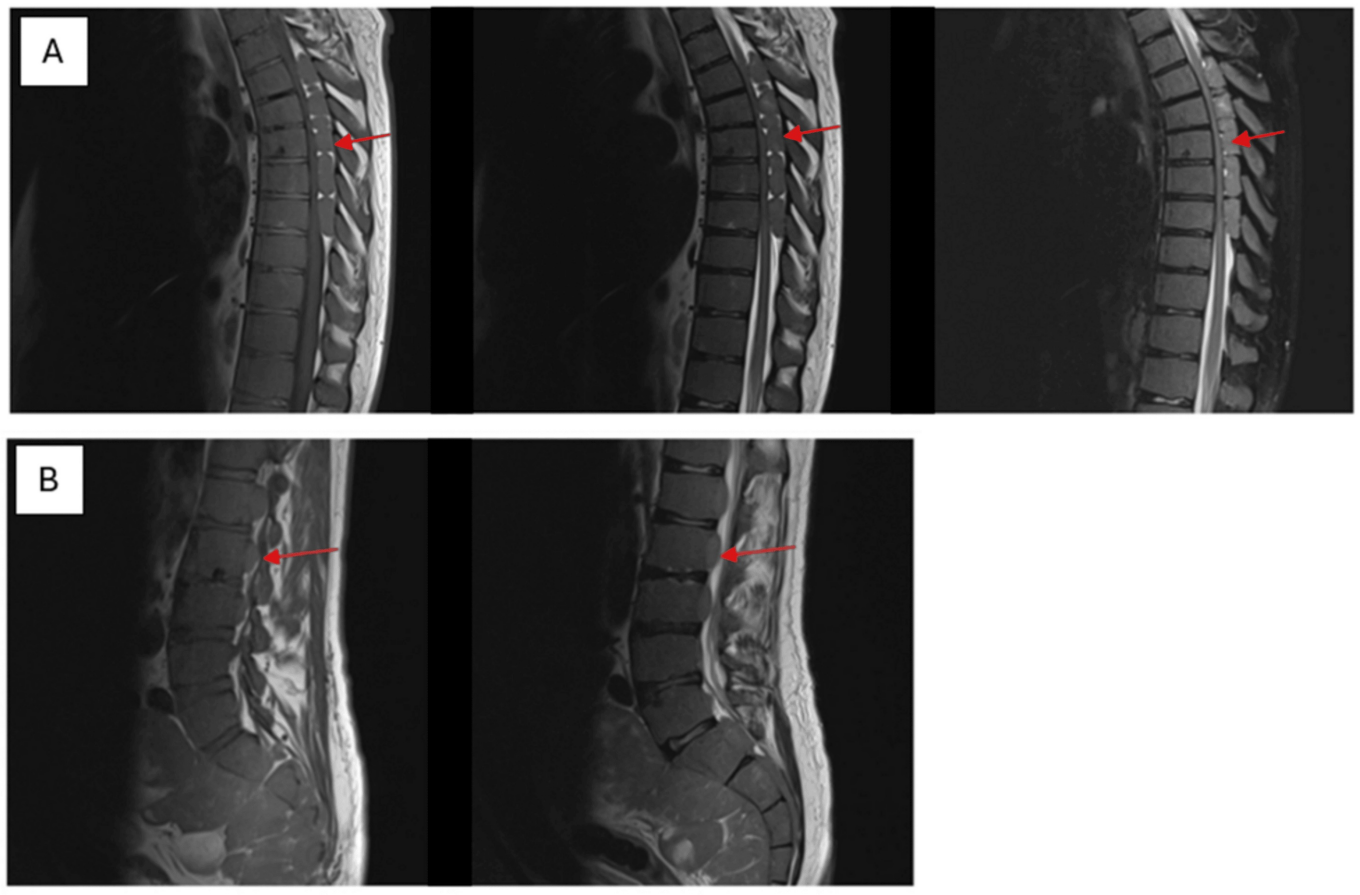
Magnetic resonance imaging (MRI) of the spine showing (A) thoracic spine and (B) lumbar spine. (A) Thoracic spine MRI (upper panels) reveals soft tissue signal abnormalities in the paravertebral region at thoracic levels, resulting in significant spinal cord compression from T4 to T8, as observed in T1-weighted, T2-weighted, and T2-weighted fat-suppressed short tau inversion recovery (STIR) sequences (red arrows). (B) Lumbar spine MRI (lower panels) displays lesions occupying the spinal canal from the S1 to S4 vertebral levels, with extension into the presacral area, resulting in notable compression of the dural sac (red arrows).

**Figure 2:**
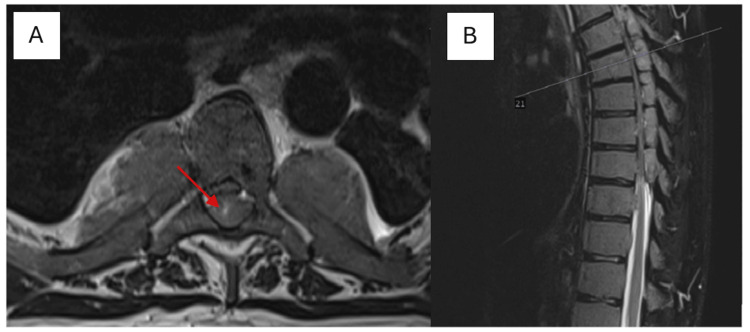
Axial (A) and sagittal (B) magnetic resonance imaging (MRI) scans of the thoracic spine. T2-weighted MRI sequences reveal soft tissue signal abnormalities within the paravertebral region at thoracic levels, resulting in severe narrowing of the spinal canal to approximately 5 mm and associated with evidence of myelopathic changes.

**Figure 3:**
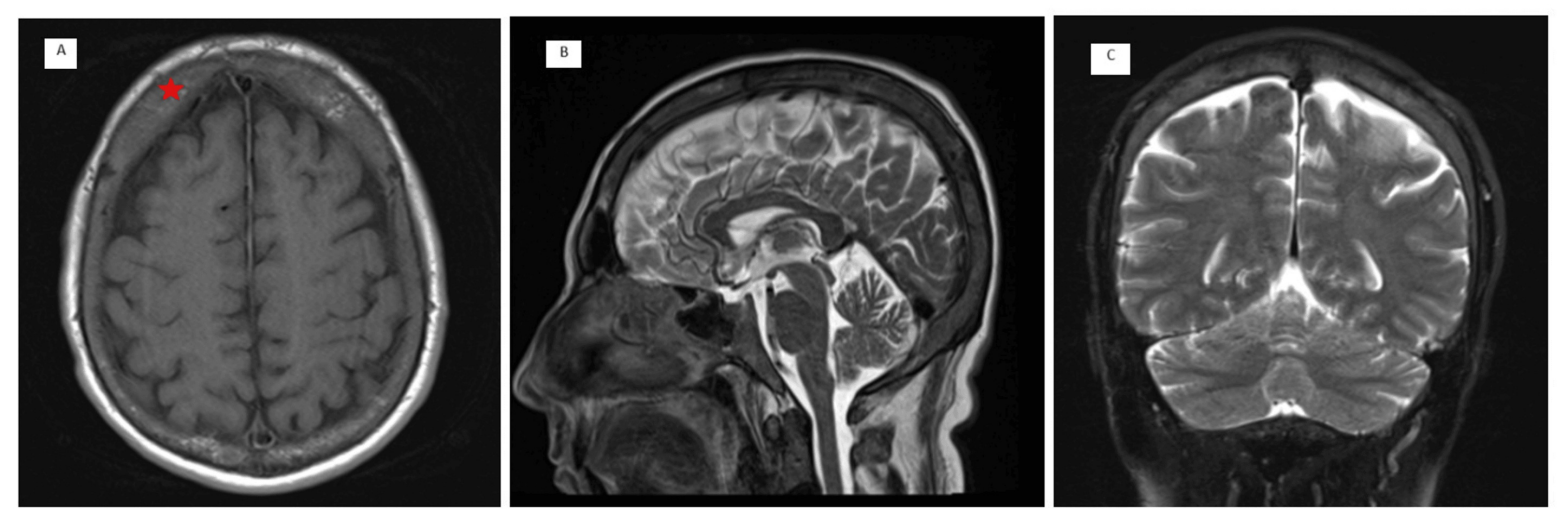
Cranial magnetic resonance imaging (MRI). Cranial MRI demonstrates marked thickening of the diploic space within the calvarial bones, most prominently in the frontal region. This feature, highlighted by a red asterisk (*), is most clearly visualized on the axial section (A), and is also appreciable on the sagittal (B) and coronal (C) planes.

The patient is a 50-year-old male with a longstanding history of TI and CML. He presented to our outpatient clinic with a chief complaint of progressive lower extremity weakness and gait imbalance, which had worsened over the past week to the point of causing significant difficulty in walking. The patient reported that his symptoms began approximately one month prior with the onset of back pain and lower back pain. Initially, these symptoms were attributed to musculoskeletal strain, and the patient sought treatment at a physical therapy and rehabilitation center. There, he was placed on a regimen of symptomatic treatment and therapeutic exercises, which provided only minimal relief. Over the subsequent weeks, he noted a gradual worsening of leg weakness and developed a significant imbalance while walking, prompting further evaluation. On examination, the patient exhibited a tandem gait disorder, characterized by unsteadiness and difficulty in maintaining balance. He displayed paraparesis with marked weakness in both lower extremities. Sensory examination revealed hypoesthesia at the L5-S1 dermatomal level and decreased deep sensation in the lower extremities. Additionally, bilateral deep tendon reflexes were absent, indicating profound neurological impairment. Non-contrast magnetic resonance imaging (MRI) of the thoracolumbar spine revealed extensive signal changes consistent with bone marrow reconversion, likely secondary to the patient's hematological conditions (Figure 1). Notably, there were soft tissue signal abnormalities in the paravertebral region at thoracic levels, indicative of EMH. These lesions filled the spinal canal from the S1 to the S4 vertebral levels and extended into the presacral area, resulting in compression of the dural sac. Lesions with similar signal characteristics were identified in the posterior epidural space extending from the T4 to T8 vertebrae, causing significant narrowing of the spinal canal to approximately 5 mm. The spinal cord exhibited increased T2-weighted signal intensity, suggestive of myelopathy due to compression (Figure 2). Cervical spine MRI without contrast revealed no detectable lesions; however, cranial MRI showed an increased diploic space within the calvarial bones, with the most pronounced changes observed in the frontal region (Figure 3).

**Figure 4:**
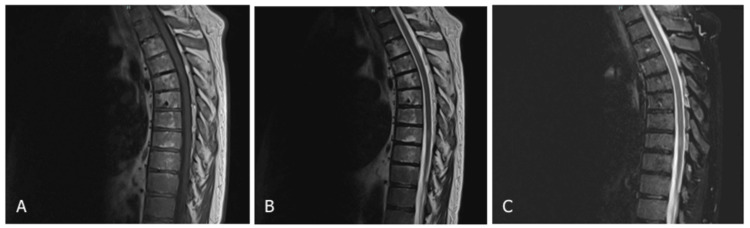
Post-radiotherapy thoracic spine magnetic resonance imaging (MRI). (A-C) Post-radiotherapy imaging demonstrated a notable reduction in the extent of compressive lesions compared to the prior neuroimaging and radiotherapy-induced thoracic spine distructions from T3 to T9.

The patient underwent external radiotherapy (RT) using the volumetric arc therapy (RapidArc; Varian Medical Systems, Palo Alto, CA, USA) technique. A total dose of 21 Gy (equivalent dose in 2 Gray fractions (EQD2) = 22.75 Gy) was delivered in seven fractions to the T3-T9 vertebrae, and a total dose of 42 Gy (EQD2 = 50 Gy) was delivered to the presacral mass extending from L5 to the coccyx. Following the initiation of treatment, the patient exhibited significant clinical improvement, which became clinically evident by the fourth week of follow-up. At the eighth week, both neurological examination and follow-up neuroimaging confirmed further recovery, demonstrating substantial regression of the compressive lesions. The final evaluation was conducted five months post-RT, at which time the patient remained clinically stable and continued to show favorable progression (Figure 4).

## Discussion

EMH is an uncommon but compensatory phenomenon seen in patients with chronic hematologic disorders, such as TI and CML [1]. These conditions often lead to ineffective erythropoiesis, creating an increased demand for blood cell production that extends beyond the marrow's capacity. As a result, hematopoiesis occurs in extramedullary sites, including the liver, spleen, and occasionally, paraspinal regions [4]. The pathophysiology of EMH involves the reactivation of hematopoietic activity in these regions, typically in response to an increased erythropoietic drive, as seen in our patient with both TI and CML [2,5]. The coexistence of these conditions presents a unique challenge, as the hyperproliferative activity associated with CML can exacerbate the hematopoietic burden, leading to increased EMH activity [6].

While EMH is often asymptomatic, it can form masses in certain areas of the body, with paraspinal EMH being particularly concerning due to the risk of spinal cord compression [4]. The thoracic spine, a common site of spinal EMH, contains residual embryonic hematopoietic tissue that can be reactivated under chronic stress conditions [7]. In this case, the formation of EMH-related masses in the thoracic region led to spinal canal narrowing, resulting in significant neurological impairment. The patient’s symptoms, which included progressive lower extremity weakness, gait imbalance, and sensory deficits, reflect the degree of spinal cord compression at multiple thoracic levels. Notably, tandem gait disorder and absence of bilateral deep tendon reflexes further underscore the severity of neurological impairment in this case.

The imaging findings in our patient align with typical presentations of spinal EMH, particularly the presence of homogeneously enhancing paraspinal and epidural masses on MRI [8]. These lesions are usually isointense to the spinal cord on T1-weighted images and hyperintense on T2-weighted images, consistent with hematopoietic tissue. The MRI findings of increased signal intensity in the T2-weighted images are indicative of myelopathy secondary to spinal cord compression [9]. Furthermore, bone marrow reconversion observed in the calvarial bones and vertebrae in this case is consistent with increased extramedullary hematopoietic activity, a common finding in patients with chronic anemia [10]. The cranial MRI showing an increased diploe distance in the calvarial bones, especially in the frontal region, and the lumbar MRI revealing medullary signal loss, support the diagnosis of bone marrow reconversion in response to the patient’s hematological conditions.

Management of spinal EMH requires a carefully individualized approach, as treatment strategies - ranging from RT and surgical decompression to hematologic therapies - must be tailored to the patient’s clinical condition, symptom burden, and underlying hematologic disorder. RT is widely considered the first-line treatment due to its non-invasive nature and rapid symptom relief, with hematopoietic tissue being highly radiosensitive and typically responsive to low doses [11]. Doses between 16 and 20 Gy, delivered in 1.8-2 Gy fractions, have been reported as effective for symptom control and lesion regression [12]. In our patient, higher radiation doses were selected due to the extensive disease burden involving both the thoracic spine and a bulky presacral mass. Dose escalation was particularly justified in the presacral region to achieve adequate local control and prevent progression. Additionally, the use of advanced techniques such as RapidArc facilitated precise dose delivery while minimizing toxicity. Although standard regimens are effective in uncomplicated EMH, higher doses, as used in our case, are supported by reports involving complex or refractory presentations [13]. Surgical intervention remains a viable option for patients with contraindications to RT or those experiencing rapid neurological deterioration. In cases of severe spinal cord compression, surgical decompression, typically via laminectomy or resection of EMH masses, can offer immediate symptom relief and prevent further neurological decline [14].

Addressing the underlying hematologic disorders is crucial in managing EMH, as it targets the root causes driving aberrant blood cell production. In TI, ineffective erythropoiesis and chronic anemia stimulate compensatory EMH [1]. While TI is often considered a non-transfusion-dependent condition, regular blood transfusions can suppress the erythropoietic drive, thereby reducing the stimulus for EMH. Clinical guidelines suggest initiating transfusion therapy in patients exhibiting significant EMH, growth retardation, or other complications related to chronic anemia [2]. However, repeated transfusions can lead to iron overload, necessitating iron chelation therapy to prevent organ damage. Chelation agents such as deferoxamine, deferiprone, and deferasirox are employed to manage iron levels, with the choice of agent tailored to the patient's needs and response to therapy [11].

In CML, the BCR-ABL fusion gene results in uncontrolled myeloid proliferation, which can contribute to EMH. Tyrosine kinase inhibitors, including imatinib, dasatinib, and nilotinib, have revolutionized CML treatment by specifically targeting the BCR-ABL oncoprotein, thereby controlling disease progression and reducing the hematopoietic burden. These therapies not only improve survival rates but also mitigate the risk of EMH by restoring effective hematopoiesis within the bone marrow [5].

This case contributes to the growing body of literature on EMH-induced spinal cord compression and highlights the importance of clinical vigilance in patients with coexisting hematologic disorders. The initial misattribution of symptoms to musculoskeletal strain led to a delay in diagnosis, highlighting a critical gap in clinical vigilance. This underscores the paramount importance of maintaining a high index of suspicion and conducting thorough neurologic and radiologic evaluations when patients with known hematologic disorders, such as TI or CML, present with new or progressive neurological symptoms. Early recognition is essential, as diagnostic delays can lead to irreversible neurologic deficits, diminished quality of life, and missed opportunities for timely intervention. Recognizing and accurately identifying serious complications like spinal EMH requires heightened clinical awareness and a thorough, holistic approach to patient evaluation. Among the most important considerations is the recommendation for long-term follow-up to monitor for EMH recurrence and to manage the underlying hematologic disorder, thereby reducing the risk of recurrent spinal cord compression and associated morbidity.

## Conclusions

EMH leading to spinal cord compression is a rare but serious complication in patients with TI and CML. Prompt recognition and treatment are imperative to prevent irreversible neurological deficits. This case highlights the necessity for an integrated and comprehensive approach to effectively address both the hematologic and neurologic dimensions of the condition.

Patients with TI and CML are at risk for EMH-related complications, including spinal cord compression. Early recognition and multidisciplinary management are crucial to prevent permanent neurological sequelae. This case underscores the importance of considering EMH in the differential diagnosis of neurological symptoms in patients with chronic hematological disorders.
